# Comparison of two mouse ameloblast-like cell lines for enamel-specific gene expression

**DOI:** 10.3389/fphys.2014.00277

**Published:** 2014-07-25

**Authors:** Juni Sarkar, Emil J. Simanian, Sarah Y. Tuggy, John D. Bartlett, Malcolm L. Snead, Toshihiro Sugiyama, Michael L. Paine

**Affiliations:** ^1^Center for Craniofacial Molecular Biology, Division of Biomedical Sciences, Herman Ostrow School of Dentistry of USC, University of Southern CaliforniaLos Angeles, CA, USA; ^2^Department of Mineralized Tissue Biology, The Forsyth InstituteCambridge, MA, USA; ^3^Department of Biochemistry, Akita University Graduate School of MedicineHondo, Akita, Japan

**Keywords:** amelogenesis, ameloblast, cell culture, LS8, ALC and biomineralization

## Abstract

Ameloblasts are ectoderm-derived cells that produce an extracellular enamel matrix that mineralizes to form enamel. The development and use of immortalized cell lines, with a stable phenotype, is an important contribution to biological studies as it allows for the investigation of molecular activities without the continuous need for animals. In this study we compare the expression profiles of enamel-specific genes in two mouse derived ameloblast-like cell lines: LS8 and ALC cells. Quantitative PCR analysis indicates that, relative to each other, LS8 cells express greater mRNA levels for genes that define secretory-stage activities (*Amelx, Ambn, Enam*, and *Mmp20*), while ALC express greater mRNA levels for genes that define maturation-stage activities (*Odam* and *Klk4*). Western blot analyses show that Amelx, Ambn, and Odam proteins are detectable in ALC, but not LS8 cells. Unstimulated ALC cells form calcified nodules, while LS8 cells do not. These data provide greater insight as to the suitability of both cell lines to contribute to biological studies on enamel formation and biomineralization, and highlight some of the strengths and weaknesses when relying on enamel epithelial organ-derived cell lines to study molecular activities of amelogenesis.

## Introduction

Dental enamel, the most highly mineralized and hardest tissue in vertebrates, is comprised of highly organized hydroxyapatite (Hap) crystallites formed by ameloblast cells (Smith, 1998). Dental enamel formation is tightly controlled by ameloblast cells whose differential activities toward enamel formation can be broadly divided into the initial secretory-stage, followed by a maturation-stage (Smith, 1998; Lacruz et al., 2013b). The enamel organ transcriptome for secretory-stage vs. maturation-stage amelogenesis is remarkably different from one another and highlights the changing cellular events orchestrated for each stage of enamel formation (Lacruz et al., 2012c, 2013b). There is a lack of available immortalized ameloblast-derived cell lines and this limits the possibilities to explore in detail the cellular events characteristic of ameloblasts through the different stages of amelogenesis, thus increasing the immediate need for animal experimentation. The few available ameloblast-like cell lines that are under investigation by the research community include mouse LS8 (Chen et al., 1992) and ALC (Nakata et al., 2003), rat HAT-7 (Kawano et al., 2002) and SF2-24 (Arakaki et al., 2012), and porcine PABSo-E (Denbesten et al., 1999) cells. LS8 cells have been used extensively to study amelogenin (*Amelx*) (Zhou and Snead, 2000; Zhou et al., 2000; Xu et al., 2006, 2007a,b) and ameloblastin (*Ambn*) (Dhamija et al., 1999; Dhamija and Krebsbach, 2001) gene promoter activities, and also fluoride uptake mechanisms and related endoplasmic reticulum (ER) stress activities (Kubota et al., 2005). LS8 (Lacruz et al., 2012a) and HAT-7 cells (Zheng et al., 2013) have been used to investigate circadian rhythm gene activities related to enamel formation. When exposed to sonic hedgehog at 100 ng/ml ALC cells are more completely differentiated into ameloblast-lineage cells, as determined by an increase in Amelx and Ambn mRNA levels (Takahashi et al., 2007). PABSo-E cells have been used to study calcium-sensing receptor activities in enamel formation (Mathias et al., 2001). PABSo-E (Duan et al., 2011) and LS8 (Shapiro et al., 2007; Lacruz et al., 2013a) cells have also been used to study endocytotic events in amelogenesis. ALC cells are noted to form calcified nodules when cultured in conditioned Dulbecco's modified Eagle's medium (DMEM) (Nakata et al., 2003), suggesting that ALC cells may be a model more suitable to the study of maturation-stage events of amelogenesis, such as ion transport associated with mineral phase maturation.

In this study we compare the molecular profiles of selected enamel-specific genes, and the ability to form calcified nodules in the two mouse-derived ameloblast-like cells: the Swiss-Webster derived LS8 cells (Chen et al., 1992) and the C57BL/6J derived and ALC cells (Nakata et al., 2003). This comparative investigation better characterizes both cell lines, and identifies some of the advantages of experimenting with one cell line in preference to the other, to focus studies to ameloblast-specific cellular phenomena such as matrix formation vs. mineral maturation.

## Materials and methods

### Animals and tissue preparation

Swiss-Webster mice were treated in accordance with Institutional and Federal guidelines. For quantitative real-time PCR and Western blot analysis, the first molars from mice at postnatal (PN) days 3, 6, and 9 were extracted and total RNA and protein recovered for analysis. These three PN time points were chosen to demonstrate the shift in ameloblast gene expression from primarily secretory-stage activities (PN3) to a stage where maturation-stage activities predominate (PN9); and a point midway (PN6) where there is a mixed population of secretory and maturation ameloblasts (Simmer et al., 2009; Hu et al., 2012). For the purpose of this study isolated first molar teeth at the three developmental stages serve as a suitable control, however RNA and protein isolated from these teeth are not only attributed by enamel organ epithelial cells but also non-epithelial cells including odontoblast and pulp cells (Jacques et al., 2014).

### Cell culture

The mouse fibroblast cell line NIH3T3 (ATCC Catalog # CRL-1658), and LS8 (Chen et al., 1992) and ALC (Nakata et al., 2003) cells were cultured in DMEM supplemented with 10% heat inactivated fetal bovine serum (FBS), in a 5% CO_2_ atmosphere at 37°C.

### RNA extraction and quantitative PCR

Total RNA was extracted from NIH3T3, LS8 and ALC cells, and from first molars of Swiss Webster mice at PN day 3, 6, and 9 using Roche High Pure RNA Extraction Kit (Roche Applied Science, Indianapolis, IN). For cell culture 7-different passages of LS8 (*n* = 7), ALC (*n* = 7), and NIH3T3 (*n* = 7) were used to extract RNA after the cells reached confluency. Extracted RNA was treated with DNAse I to remove all residual genomic DNA. RNA samples were further purified with DNase Inactivation Beads (Cat # AM1907, Ambion, NY, USA) according to the manufacturer's protocol to remove contaminating genomic DNA. 5 μg of total RNA was reverse transcribed to first strand cDNA using a cDNA Synthesis Kit (Takara Bio, Clontech, Mountain View, CA). The cDNA was analyzed using mouse specific Actb (a.k.a. beta actin or β-actin) primers to confirm the absence of contaminating DNA from the isolation process (Table 1). The PCR conditions applied were: 94°C for 30 s; 55°C for 30 s; and 72°C for 1 min for 30 cycles. The PCR products were electrophoresed in a 2% agarose gel, stained with ethidium bromide, and visualized under ultraviolet light.

**Table 1:** **Primers used in this study**.

**Gene ID**	**Forward primer 5′-3′**	**Reverse primer 5′-3′**	**Product (bp)**
*Amelx*	GTCACCTCTGCATCCCATG	TTCCCGCTTGGTCTTGTC	136
*Ambn*	TGAGCCTTGAGACAATGAGAC	AAAGAGTTATGCGGTGGGAG	133
*Enam*	TATGGTCTTCCACCAAGGAA	TAGGCACACCATCTCCAAAT	181
*Mmp20*	CACCTCACAAGCCATCTATCC	GAAGCTCCTTTCCCAACATTG	81
*Amtn*	GCACATACTCTCCCGTTCAC	AAGATTTGGGAGGCTAACGG	144
*Odam*	TTGACAGCTTTGTAGGCACA	GACCTTCTGTTCTGGAGCAA	197
*Klk4*	CAACTAAAGAATGGGAAACTGCC	GAGTCCTTTTGGTCCTGTCC	143
*Actb*	CTGGCACCACACCTTCTACAATG	GATGTCACGCACGATTTCCCTC	382

For real-time PCR, mouse specific primers of *Amelx, Ambn, Enam, Mmp20, Amtn, Odam, Klk4*, and *Actb* were used (Table 1). PCR was accomplished with iQ SYBRGREEN Supermix (BioRad Laboratories, Hercules, CA) according to the manufacturer's instructions. Expression quantity was analyzed by ABI PRISM 7500 (Applied Biosystems, Grand Island, NY). The PCR conditions were 94°C for 1 min followed by 95°C for 15 s and 62°C for 34 s for 40 cycles. All samples were run in triplicate. All data points were normalized to *Actb*.

### Western blot analysis

Total protein was extracted from cell pellets at either 3 or 7 days after passage, and from mouse first molars (maxillary and mandibular) at post natal (PN) days −3, −6, and −9, using ice-cold RIPA lysis buffer (Pierce Biotechnology, Inc., Rockford, IL), supplemented with Halt Protease Inhibitor Cocktail (Thermoscientific, Rockford, IL). For protein extraction from mouse molars, the samples were homogenized manually with a pestle for 40 s. Samples were then cleared by centrifugation and protein concentration quantified using the Bicinchoninic Acid (BCA) assay (Pierce Biotechnology). Ten microgram of total protein from mouse molars and 20 μg of total protein from cell culture were size resolved on denaturing 12% Tris–HCl polyacrylamide gels by SDS gel electrophoresis and transferred to nitrocellulose membranes to detect the enamel proteins. Antibodies used were: mouse-derived anti-Gapdh (GA1R, Thermo Scientific); chicken-derived anti-Amelx (Shapiro et al., 2007); rabbit-derived anti-Ambn (sc-50534, Santa Cruz Biotechnology, Dallas, TX); rabbit-derived anti-Amtn (Lacruz et al., 2012b); and rabbit-derived anti-Odam (Moffatt et al., 2008). Secondary antibodies used were: horseradish peroxidase labeled goat-derived anti-mouse (Cat # 32430, Pierce Biotechnology); goat-derived anti-rabbit (Cat # 32460, Pierce Biotechnology); and rabbit-derived anti-chicken (Cat # 31401, Pierce Biotechnology). Labeled protein bands were detected with an Enhanced ChemiLuminescence (ECL) detection system (Pierce Biotechnology).

### Alizarin Red S staining

5 × 10^4^ cells/well were inoculated in 12 well plates. Cells were fixed with 10% formaldehyde for 20 min and stained with 1% Alizarin Red S provided in the Osteogenesis Quantification Kit (Millipore, Billerica, MA) for detection of mineral under phase contrast microscope. The cells were evaluated for Alizarin Red stain at 1-, 3-, 7-, 14-, 21-, and 28-days post inoculation.

### Quantitative analysis of Alizarin Red staining

Stained monolayer cells fixed in the above method were detached using 10% acetic acid as described in manufacturer's protocol of the Osteogenesis Quantification Kit (Millipore). Cells were recovered by mechanically scrapping off the well, and the cells recovered into a microcentrifuge tube and vortexed vigorously. Tubes were heated at 85°C for 10 min and stored on ice for 5 min. The cell suspension was cleared of debris by centrifugation at 20,000 × g for 15 min. Four hundred microliter of supernatant was transferred into new microcentrifuge tube and pH was neutralized with 150 μL of 10% ammonium hydroxide. One hundred and fifty microliter of this supernatant was transferred into an opaque walled transparent bottom 96-well plate, and the optical density of the suspension was measured by absorption at 405 nm. Alizarin Red stain incorporated into the cells was quantified using a standard curve generated as per the protocol provided by the manufacturer. A standard plot for Alizaren Red concentration (range = 2.0 mM–0.47 μm) vs. OD_405_ was applied to quantify the unknown samples.

### Statistical analysis

The data were analyzed for statistical significance using Student's *t*-test and values of *p* < 0.05 were considered statistically significant.

## Results

### Enamel-specific mRNA expression in LS8, ALC, and mouse molars

**Figure 1:**
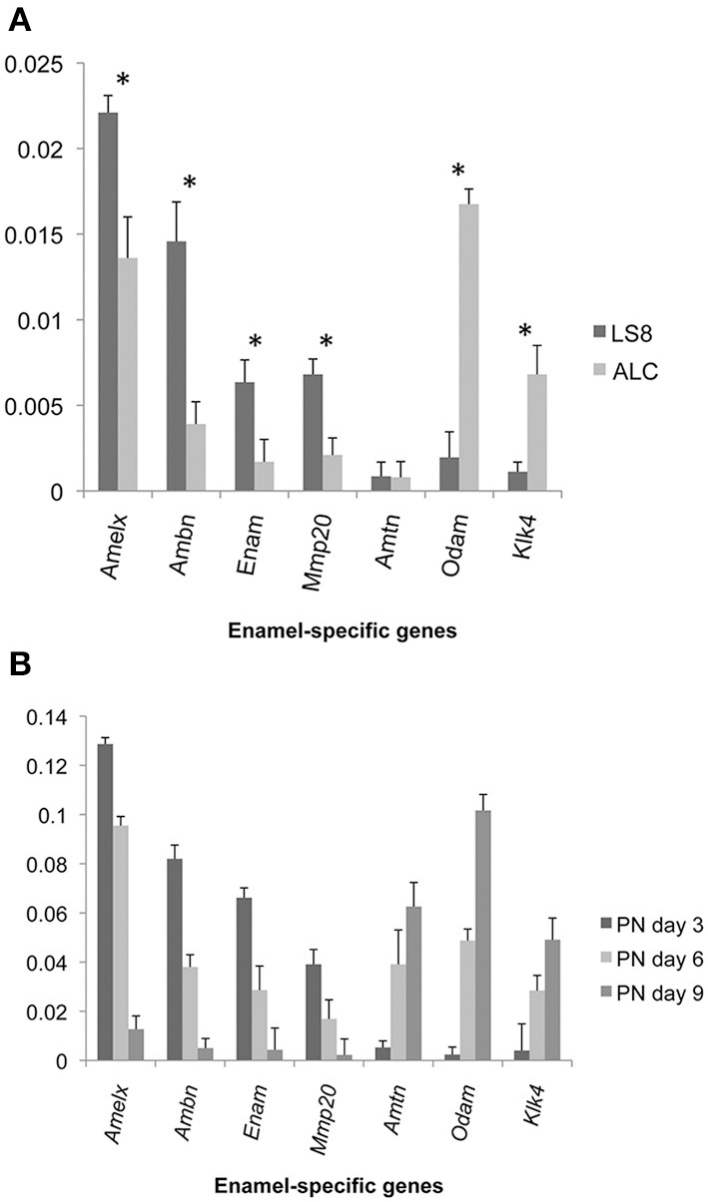
**Expression of enamel-specific genes in LS8 and ALC cells, and mice first molars at post natal (PN) days 3, 6, and 9**. **(A)** Results of real-time PCR analyses for LS8 and ALC. The expression of each enamel-specific gene was compared within the two cell lines, and normalized to β-actin. mRNA transcript levels were compared between LS8 and ALC for Amelx, Ambn, Enam, Mmp20, Amtn, Odam, and Klk4. Average expression levels, and standard error of deviation, were calculated from seven independent real-time PCR amplifications (each run in triplicate; *n* = 7). Expression levels relative to Actb expression are graphed. With the exception of Amtn, there was a statistically significant difference in expression (*p* < 0.05) between both cell lines. **(B)** The expression of each gene with the enamel genes is compared from three stages of development; PN3, 6, and 9, and normalized to Actb levels. For each gene examined there were statistically significant (^*^p < 0.05) expression level differences noted between PN3 and PN6, PN3 and PN9, and PN6 and PN9 days. Expression levels relative to Actb expression are graphed.

Enamel gene-specific primers (Table 1) were used to compare mRNA transcript levels in ameloblast-like (LS8 and ALC) cells, and mouse molars (PN days 3, 6, and 9). Messenger RNA expression levels in LS8 and ALC, relative to β-actin, were also compared (Figure 1A). Higher mRNA levels for *Amelx, Ambn, Enam*, and *Mmp20* were noted in LS8 cells, while ALC cells expressed higher mRNA levels for *Odam* and *Klk4*. Expression of *Amtn* in both cell lines was negligible. These expression levels were then compared to mRNA transcripts in mouse first molars isolated at PN3, 6, and 9 (Figure 1B). This *in vivo* derived molar data clearly demonstrated a shift from a more dominate secretory function, with high levels of expression for *Amelx, Ambn, Enam*, and *Mmp20* at PN3, to a more dominant maturation-stage gene expression profile, with the highest levels of *Amtn, Odam*, and *Klk4* seen at PN9. The data may indicate that ALC gene expression profiles represent a later stage of gene expression associated with enamel maturation, when compared to the gene expression profiles of LS8 cells that may represent better a secretory function.

### Enamel-specific protein expression in LS8, ALC, and mouse molars

**Figure 2:**
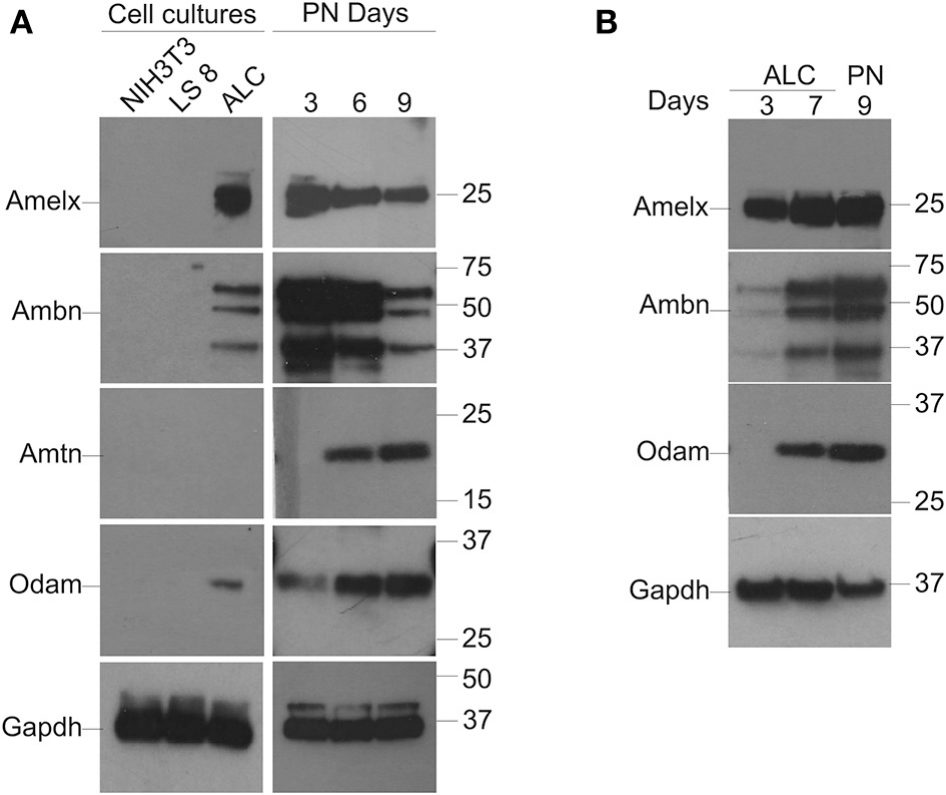
**Protein expression in LS8 and ALC cells, and *in vivo* mouse molars. (A)** Groupings to left: Western blot analysis of the expression of Amelx, Ambn, and Odam is noted in ALC cells only. LS8 cells did not appear to express the enamel proteins examined. No Amtn expression was noted in either enamel organ cell line. NIH3T3 (fibroblasts) cells are used here as a negative control. All cells cultured for 7 days. Groupings to right: Protein from PN3, 6, and 9 mouse first molars were examined to confirm the suitability of each antibody to assess mouse-derived enamel proteins; and also to illustrate the decreasing levels of Amelx and Amtn from PN3 to PN9, and the increasing levels of Amtn and Odam over the same time course. **(B)** A second series of Western blots were completed with ALC cells isolated at days 3 and 7 after passage, and also from PN9 mouse molars to directly compare protein levels for Amelx, Ambn, and Odam in ALC cells when compared to *in vivo* derived tooth tissues. Glyceraldehyde-3-phosphate dehydrogenase (Gapdh) was used as a loading (normalizing) control.

Western blot analysis for Amelx, Ambn, Amtn, and Odam was performed on protein extracts isolated from NIH3T3, LS8, and ALC cells as described in Materials and Methods and for protein lysates from PN3, 6, and 9 molars (Figure 2A). Gapdh was applied as a normalizing control. Suitable antibodies for Western blot analysis of Enam, Mmp20, and Klk4 protein were not immediately available thus their expression profiles were not examined. Unlike the mRNA expression profiles that could be appreciated in both LS8 and ALC cells, protein for Amelx, Ambn, and Odam was evident only in ALC cells (Figure 2A). No protein gene expression associated with enamel matrix was detected in LS8 cell lysates, or by the NIH3T3 cells used as a negative control. In the case of the *in vivo* derived molar PN3, 6, and 9 samples, the selected protein expression profiles reflected the levels observed for the mRNA expression profile, with the highest expression levels for Amelx and Ambn apparent at PN3, and the highest levels of Amtn and Odam expression apparent at PN9.

A second series of Western blots were performed using protein extracts from ALC cells cultured for 3- and 7-days after passage, alongside protein extracted from PN9 first molars (Figure 2B) with Gapdh applied as the normalizing factor. This data suggest that ALC cells show robust levels of Amelx, Ambn, and Odam after 7-days in culture when compared to these same ALC cells cultured for 3-days.

### Quantitative analysis of biomineralization ability of ALC cells

**Figure 3:**
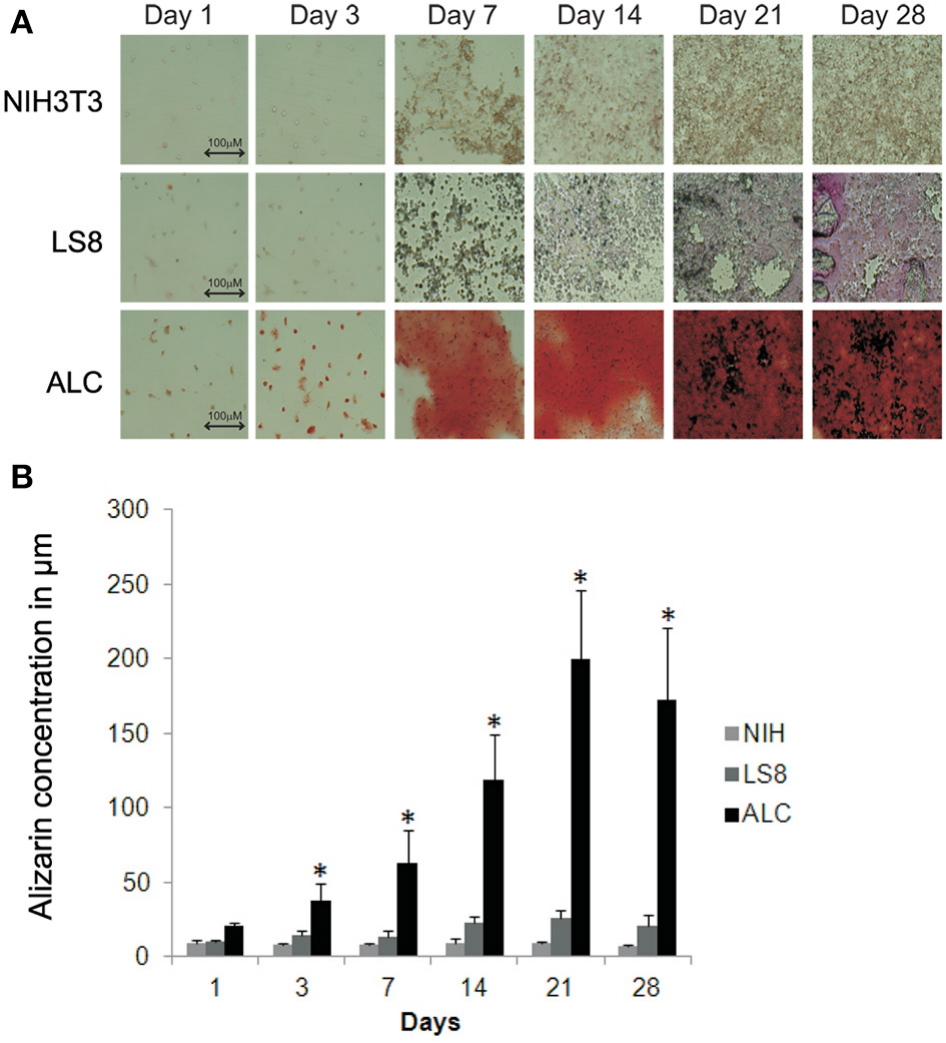
**Biomineralization assay for ALC and LS8 cells. (A)** Alizarin Red staining was detected at levels significantly higher than background at day 3 post inoculation, and this is characteristic of calcified nodule formation. In ALC cells, stain accumulation increased over a period of 21 days, with no significant change beyond 21-days. Only background level staining was observed in LS8 cells, when compared to NIH3T3 cells (negative control) at day 1 and also over this same time period. Scale bar 100.0 μm. **(B)** Alizarin Red stain in NIH3T3, LS8, and ALC cell culture were quantified using Osteogenesis quantification kit at OD_405_. Graph shows significant increase in red staining in ALC cells only. Significance indicated by ^*^ for *p* < 0.05 for differences in individual cell lines.

LS8 and ALC cells, and NIH3T3 cells (negative control), were compared to assess their abilities to form calcified nodules in a defined culture condition (DMEM supplemented with 10% FBS) without additional growth factors. ALC cells showed positive Alizarin Red S staining, which became apparent at day-3 and increased over the 28-days duration of culture (Figure 3A). NIH3T3 cells showed negligible Alizarin Red reactivity, and LS8 cells revealed only modest staining at the end of the 28-days. Alizarin Red S staining was quantified by extrapolating from a standard curve of concentrations measured at 405 nm. Graphical representation of the solubilized Alizarin Red S stain from each cell culture indicated a progressive, and highly significant, increase in calcified nodule formation for ALC cells only, reacting a maximum staining at day 21 (Figure 3B).

## Discussion

This current study has focused on comparing two mouse ameloblast-like cell lines (LS8 and ALC) with respect to their expression of enamel specific genes as mRNAs and proteins. The purpose of the comparison is to determine if two “well-established” and “widely-studied” ameloblast-like cell lines could be distinguished from each other, and if one line could to be judged as more suitable to study certain molecular aspects of amelogenesis. Based on mRNA gene expression profiles LS8 cells exhibit higher levels of *Amelx, Ambn*, and *Enam*, and *Mmp20* mRNAs, while ALC cells show noticeably higher levels of *Amelx, Odam*, and *Klk4* mRNAs. Both cell lines show significant levels of *Amelx* transcripts, and as such, both should be suitable to investigate *Amelx* gene transcriptional regulatory activities. These transcriptome profile data also suggest that LS8 cells may be preferable in a study of transcriptional regulation for *Ambn, Enam*, or *Mmp20*, while ALC cells may be the more appropriate cell line to study transcriptional activities related to *Odam* and *Klk4*.

From the quantitative PCR analysis we may conclude that, relative to each other, LS8 cells are less differentiated than are the ALC cells because of the higher mRNA levels seen for gene transcripts related to secretory-stage activities (*Amelx, Ambn, Enam*, and *Mmp20*). This conclusion is also supported by the fact that ALC cells show higher levels of *Odam* and *Klk4* mRNAs, and also that ALC cells form calcified nodules in culture while LS8 cells do not, and that calcification/mineralization of the enamel matrix is characteristic of the maturation-stage of amelogenesis, *in vivo*. However, our protein analysis presents an alternative body of data which suggests that translational events of the mRNA expressed by LS8 and ALC cells are very different, as Amelx and Ambn (secretory-stage proteins), and Odam (maturation-stage protein) were all detected in ALC cells only.

This study clearly has its limitations and could be further expanded. For example we have been very selective in studying only a small number of enamel-specific genes at the message level (*Amelx, Ambn, Enam, Mmp20, Amtn, Odam*, and *Klk4*), and from these, only four (Amelx, Ambn, Amtn, and Odam) were investigated at the protein level. Performing a whole genome transcriptome analysis with microarray technology would help to determine if other events that define amelogenesis are apparent in either cell line. A second experiment of value would be to establish if the secretory activities observed in ameloblasts *in vivo* are active in cell culture, and which of these enamel proteins are secreted into the culture medium. For example, even though Amelx protein was not evident from the disrupted LS8 cells, there may be evidence for the protein in the medium. All the enamel proteins studied here are secreted products, so an investigation of ameloblast secretory dynamics would also be of value to better define each of the cell lines' biological characteristics. In any case, this study is the first of its kind to compare two ameloblast-like cell lines side by side.

## Conclusion

When studying biological phenomenon in health and disease, investigators are limited by the resources they have at hand. Small animal models (e.g., mice and rats) are often used as surrogates for humans, and restrictions are in place to limit animal experiments such that statistically significant data is collected from the most appropriate species using minimal animal numbers. Cell culture allows for experimentation on cells derived from animal sources, and is a valuable tool to investigate many aspects of cell and molecular biology. The use of recovered canonical proteins or recombinant produced proteins assembled in an artificial environment is another alternative to investigate complex biological processes in a systematic manner, *in vitro*. What appears still to remain a limitation to these types of studies is the availability of suitable antibodies for each protein of interest. Finally, while different approaches to study certain biological phenomenon each have their strengths and weaknesses, each offer invaluable insight into biology at the molecular level. Creating a framework of knowledge from *in vitro* cell or protein models permits well-designed and focused studies to be conducted on whole animals to validate outcomes and extend our capacity to mimic Nature.

### Conflict of interest statement

The authors declare that the research was conducted in the absence of any commercial or financial relationships that could be construed as a potential conflict of interest.

## Abbreviations

Actb: beta actin
Gapdh: glyceraldehyde-3-phosphate dehydrogenase
Amelx: amelogenin
Ambn: ameloblastin
Enam: enamelin
Mmp20: matrix metallopeptidase 20
Amtn: amelotin
Odam: odontogenin ameloblast associated
Klk4: kallikrein related-peptidase 4
DMEM: Dulbecco's modified Eagle's medium
PN: post natal
RIPA buffer: radio-immunoprecipitation assay buffer
BCA: bicinchoninic acid
SDS: sodium dodecyl sulfate.
